# FHP-ClsNet: a model for fetal head position classification based on intrapartum ultrasound

**DOI:** 10.3389/fmed.2026.1830543

**Published:** 2026-05-20

**Authors:** Shengjie Wu, Yating Hong, Shijie Zhang, Ju Tang, Jing-Ming Guo, Yuling Fan, Ping Li, Peizhong Liu, Jihong Song, Shunlan Liu

**Affiliations:** 1 College of Engineering, Huaqiao University, Quanzhou, Fujian, China; 2 Department of Gynecology and Obstetrics, Fujian Medical University Affiliated First Quanzhou Hospital, Quanzhou, China; 3 Department of Ultrasound, Second Affiliated Hospital of Fujian Medical University, Quanzhou, Fujian, China; 4 School of Medicine, Huaqiao University, Quanzhou, Fujian, China; 5 Department of Electrical Engineering, National Taiwan University of Science and Technology, Taipei, Taiwan; 6 School of Nursing, Fujian Medical University, Fuzhou, Fujian, China

**Keywords:** attention mechanism (AM), deep learning, fetal head position, image processing, intrapartum ultrasound

## Abstract

**Objectives:**

This study aims to develop an advanced deep learning model for accurate classification of fetal head positions (occiput anterior [OA], occiput posterior [OP], and occiput transverse [OT]) in intrapartum ultrasound images, which is crucial for labor management and delivery outcome prediction.

**Methods:**

We proposed FHP-ClsNet, a model integrating CBAM, ECA, and PSA attention mechanisms with an AIFI feature interaction module, based on YOLOv8 architecture. The study utilized 69 annotated intrapartum ultrasound videos divided into training, validation, and test sets. Data augmentation strategies including random flip, geometric transformation, and Mosaic enhancement were applied. Model performance was evaluated using accuracy (ACC), precision-recall area under curve (PR-AUC), receiver operating characteristic area under curve (ROC-AUC), and kappa coefficient.

**Results:**

The model achieved exceptional performance metrics: 0.8961 ACC, 0.8899 PR-AUC, 0.9401 ROC-AUC, and 0.8434 kappa score. Ablation experiments confirmed the significant contributions of attention mechanisms and feature interaction modules. Compared to baseline models, our model improved ACC by 1.6% and kappa by 3%. In video classification, it correctly identified 86/109 cases, outperforming other models.

**Conclusion:**

FHP-ClsNet demonstrates high accuracy in fetal head position classification from static ultrasound images, showing potential to assist clinical decision-making in labor management, though further improvements are needed for video sequence processing.

## Introduction

Intrapartum Ultrasound (IPUS), an ultrasound technique utilized during labor, originated in the 1950s and has become widely used clinical practice as technology has advanced (1–4). The Society of Ultrasound in Obstetrics and Gynecology (ISUOG) guidelines emphasize its importance in enhancing clinical management and provide recommendations for appropriate use (5). Fetal position, which refers to the fetus's position and posture relative to the maternal pelvis, significantly impacts the fetus's health and delivery mode (6, 7). The occiput anterior (OA) position facilitates natural delivery, while abnormal positions like occiput posterior (OP) or occiput transverse (OT) may increase dystocia and perinatal complications risks. Studies show that labor durations are longer in lateral and occiput posterior groups compared to the anterior group (8). Furthermore, the incidence of persistent OP and OT positions varies across labor stages, occurring in approximately 5%−12% and 3%−8% of cases, respectively (9–12). Timely detection and appropriate interventions can correct abnormal positions and improve natural delivery rates (13).

In traditional obstetric practice, clinicians mainly rely on the traditional method of transvaginal digital palpation to assess the position of the fetus. Digital palpation is essentially a method of palpation that highly relies on the personal experience and subjective feeling of the operator. Although intrapartum ultrasound can greatly improve the above problems, the interpretation of its images is still highly dependent on the professional knowledge and clinical experience of sonographers. It takes a long time to train a doctor to be skilled in interpreting intrapartum ultrasound images, and there are certain time delays and subjective differences in manual reading. In order to free physicians from heavy image analysis and achieve rapid, standardized, and reproducible fetal head position interpretation, it is necessary to develop automated fetal head position classification models.

Deep learning models are increasingly applied in medical ultrasound image analysis, with potential in tasks like standard plane detection and anatomical structure analysis (14–16). Recent studies have focused on pose estimation, preterm birth prediction, and maternal-fetal ultrasound classification (17, 18). Algorithms for fetal head position identification have been developed, achieving high accuracy (19, 20). Fet-Net, introduced in 2023, detects fetal head position in MRI with high accuracy (21). In 2024, a model for fetal head position identification demonstrated impressive accuracy and specificity (22).

Most existing fetal head position classification studies rely on standard classification networks or simple CNN architectures. These models face two major challenges when processing ultrasound images: (1) the fuzzy texture and low feature contrast of intrauterine ultrasound images make it difficult for standard models to capture subtle anatomical differences; (2) Most existing works ignore the modeling of long-range dependencies within feature maps. The contribution of this paper is to introduce YOLO architecture into the task of fetal head position classification, and integrate three heterogeneous attention mechanisms, CBAM, ECA, and PSA, with the feature interaction module of AIFI. This design specifically aims at the precise localization and feature enhancement of key anatomical landmarks (such as orbit and thalamus) in ultrasound images, which solves the problems of limited receptive fields and insufficient information interaction of traditional CNN, thereby improving the interpretability of the model while maintaining high accuracy.

## Materials and methods

**Figure 1:**
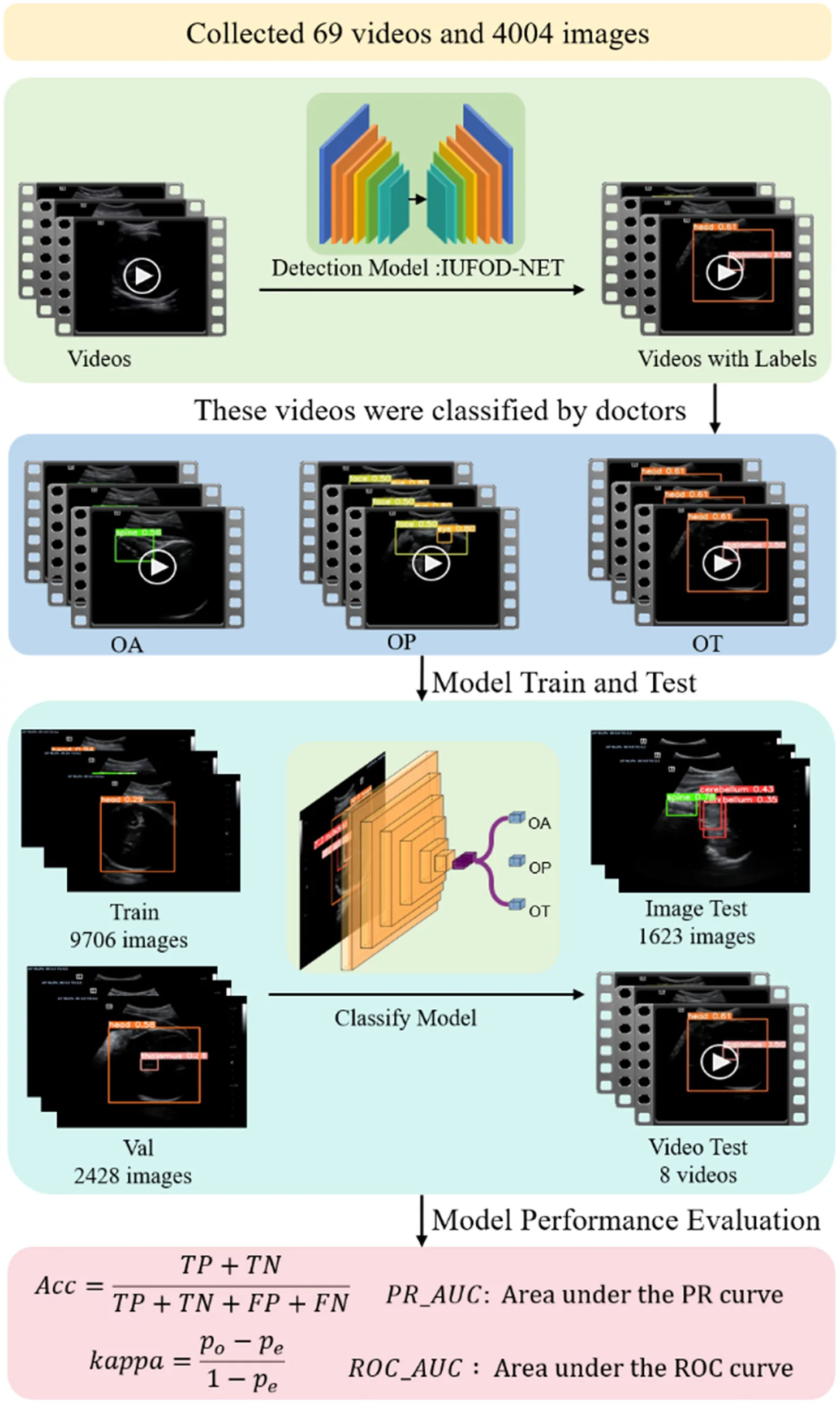
Main workflow.

The overall workflow of this study is shown in Figure 1. Firstly, the clinical experts included the pregnant women undergoing intrapartum ultrasound examination and obtained the fetal intrapartum ultrasound video. Then, the key anatomical structures and organs in the fetal ultrasound video that assisted the judgment of fetal head position were annotated by the fetal key organ detection model of intrapartum ultrasound previously studied by the project team. The videos were accurately classified by professional doctors, and then the deep learning fetal head position classification algorithm was trained in the training set and the test set respectively. Finally, the model performance was evaluated and summarized.

### Participants characteristics

In our retrospective study, pregnant women with an average age of 27 years old and in labor (37+ weeks) were enrolled from January 2023 to March 2024 in the Second Affiliated Hospital of Fujian Medical University. A total of 178 videos and 4,004 images were collected for this study. This study was approved by the Medical Ethics Committee (Ethical approval No. 2023656), and all patients signed informed consent.

### Dataset construction method

**Figure 2:**
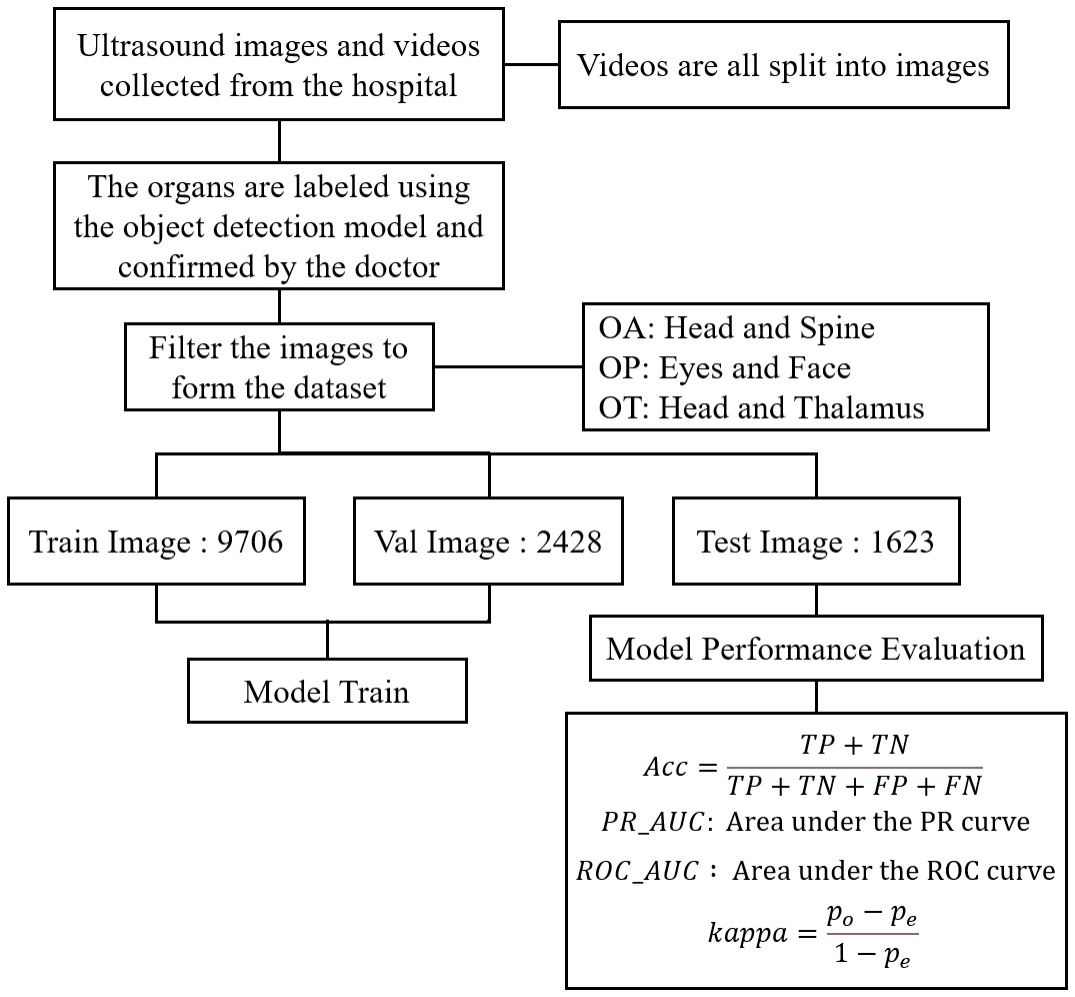
Data set construction process.

According to the guidelines of intrapartum ultrasound, the landmarks of the occiput posterior position were the two orbits. The landmarks of occiput transverse position were the midline echo, thalamus, and choroid plexus. The landmarks of the occiput anterior position are the occiput and spine. Combined with the statistical results of organ detection and the landmarks of different positions proposed in intrapartum ultrasound, the final dataset was constructed according to the following methods: for each video, the organs appearing in each frame of the ultrasound video were identified and labeled by professional physicians, and the category label of each image was given. Pictures were classified into 3 categories based on the orientation of the occiput anterior (OA), occiput posterior (OP), and occiput transverse (OT). Images featuring the head and spine were selected to represent the occiput anterior position; those featuring the eyes and face were selected for the occiput posterior position; and those featuring the head and thalamus were selected for the occiput transverse position (Figure 2).

A patient-based partitioning strategy was used in this study. We collected 4,004 images (3,886 pregnant women), 178 videos (167 pregnant women), and randomly selected 120 pregnant women from the pregnant women with video data for the final model test. We did not participate in the training of the model, and the rest of the data was divided into the training process for model training (the videos used for training will be segmented into pictures). This means that all video frames from the same patient will only appear completely in one dataset and will never appear in both the training and testing sets.

We used the collected data to construct a training set and a test set according to the above method, and obtained a total of 9,706 images as the training set and 2,428 images as the validation set (both training set and validation set belong to the training process. The training set was used for model forward propagation, and the validation set was used for model back propagation and parameter fine-tuning. The training set and validation set were randomly divided into all the data used in the training process according to 8:2). The 1,623 pictures (Table 1) obtained by segmentation of 11 videos were used as the picture test set, and 109 videos were used as the video test set (Table 2).

**Table 1:** Comparison of classification performance across different models.

Model	PR-AUC	ROC-AUC	Accuracy	Kappa
Efficientnet-b0	0.5026	0.6873	0.5136	0.2199
Densenet-121	0.7556	0.8246	0.6533	0.4612
Resnet-50	0.8205	0.8856	0.7795	0.6644
ConvNeXt	0.8283	0.8823	0.7268	0.5838
YOLOv5-cls (224)	0.8576	0.9101	0.8132	0.7111
YOLOv5-cls	0.8343	0.9158	0.8743	0.7987
YOLOv8-cls (224)	0.8309	0.9027	0.8014	0.6941
YOLOv8-cls	0.8719	0.9282	0.8806	0.8169
Ours model	**0.8899**	**0.9401**	**0.8961**	**0.8434**

The bold value represents the best result in the comparison.

**Table 2:** Results of different models for video classification.

Model/doctor	Correctly classified
Ours model	86
EfficientNet-b0	75
DenseNet-121	81
ResNet-50	78
YOLOv5-s	81
YOLOv8-l	83
Midwife	82
Sonographer	90

### Deep learning method

**Figure 3:**
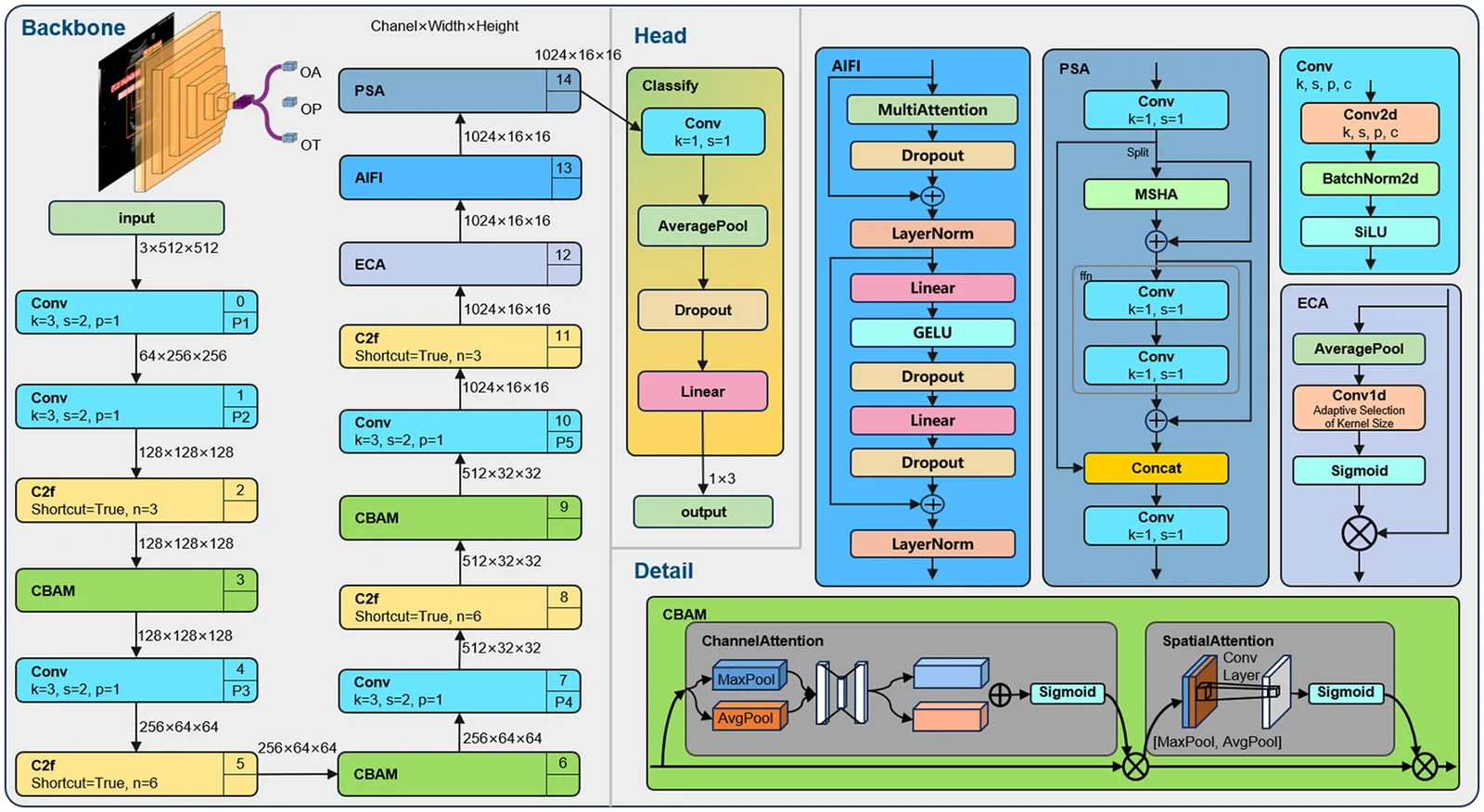
Model structure diagram.

In this study, we took YOLOv8 as the baseline model, and after many experiments, we designed a deep learning model for classifying the fetal head position (occiput anterior, occiput posterior and occiput transverse) of the pictures in the video of intrapartum ultrasound. The model structure is shown in Figure 3. The model structure mainly includes three parts: input, backbone and classify head.

#### Input

The input part is mainly responsible for data enhancement of the image to improve the sample quality and the generalization ability of the model. In this part, the input image is first processed by batch normalization to adjust the image to 512 x 512 pixels, followed by random flip (horizontal and vertical), geometric change (rotation, translation), color space transformation (brightness, contrast, saturation random adjustment), and data enhancement strategies such as Mosaic enhancement. Data augmentation generates more training samples by changing the input data to reduce the excessive dependence of the model on specific features and avoid overfitting.

#### Backbone

The backbone network is responsible for feature extraction, which adopts convolution and deconvolution layers, combines residual connection and bottleneck structure, and adds attention mechanism to strengthen the expression of important features. Backbone consists of six modules: Conv, C2f, CBAM (23), EMA (24), PSA (25), and AIFI (26). Compared with the YOLOv8 baseline model, this study added CBAM, EMA, PSA attention mechanism modules and AIFI modules to improve the feature extraction efficiency and model performance. Among them, the Conv module consists of convolutional layer, batch normalization layer and SiLU activation function, which is responsible for feature extraction. The C2f module is used to aggregate multiscale information by concatenating the output of different Bottleneck modules and the original feature maps. The CBAM attention mechanism combines channel attention and spatial attention, generates channel descriptors by global average pooling and global maximum pooling, and learns attention weights through MLP to realize feature recalibration of channel and spatial dimensions. The ECA module captures the dependencies between channels by one-dimensional convolution to improve the performance of the network. The PSA module, designed for fine-grained pixel-level tasks, reduces information loss by maintaining high resolution and employs nonlinear functions to enhance feature representation capabilities. Based on the feature interaction module of attention mechanism, the AIFI module enhances the feature extraction ability in object detection and focuses on processing high-level image features through self-attention mechanism.

#### Classify head

The final classification task consists of a series of convolutional layers that output a fixed-length vector, each element representing a category score. The score vector was transformed into a probability distribution by the softmax function to obtain the final classification result.

### Evaluation index

To evaluate the model performance, we used the trained model to predict the picture test set and the video test set. In the test set of pictures, Accuracy: the percentage of predicted correct results in the total sample; Area under the precision-recall curve (PR-AUC): the precision at different recall points was averaged to reflect the average precision of the model; receiver operating characteristic (ROC) curve area (ROC-AUC): indicates the ability of a model to distinguish between positive and negative cases. The value is between 0 and 1, and the larger the value is, the better the performance of the model. Kappa coefficient: a consistency test index based on the confusion matrix to measure the classification effect. The calculation formulas for these indicators are shown in the Table 3 below. Through these evaluation indexes, the performance of the model is comprehensively evaluated to ensure the validity and reliability of the model in practical application.

**Table 3:** The calculation formula for the evaluation indicators.

Metrics	Equation
ACC	$\frac{TP+TN}{TP+TN+FP+FN}$
P	$\frac{TP}{TP+FP}$
R	$\frac{TP}{TP+FN}$
PR-AUC	$\overset{m}{\underset{i=1}{\sum }}\left(R_{i}-R_{i-1}\right)\times$ P_*i*_
TPR	$\frac{TP}{TP+FN}$
FPR	$\frac{TP}{TP+TN}$
ROC-AUC	$\frac{1}{2}\overset{n-1}{\underset{i=1}{\sum }}\left({\text{TPR}}_{i}-{\text{TPR}}_{i-1}\right)\times \left(FPR_{i}+FPR_{i+1}\right)$
Kappa	$\kappa =\frac{P_{o}-P_{e}}{1-P_{e}},P_{o}=\frac{\overset{k}{\underset{i=1}{\sum }}O_{ii}}{N},P_{e}=\overset{k}{\underset{i=1}{\sum }}\frac{R_{i}\times C_{i}}{N^{2}}$

### Experiment settings

The experiment was conducted on a computer running Windows 11 with the following hardware configuration: the 11th Gen Intel(R) Core (TM) i7-11700 served as the CPU, 16GB of memory, and an NVIDIA GeForce RTX3060 with 12GB of GPU memory. The programming environment used is Python3.9, and the deep learning framework used in the experiment is Pytorch 2.3. In addition, the Settings of learning rate, optimizer and other parameters in the experiment were based on the baseline model YOLOv8.

## Results

### Train results and classify performance

**Figure 4:**
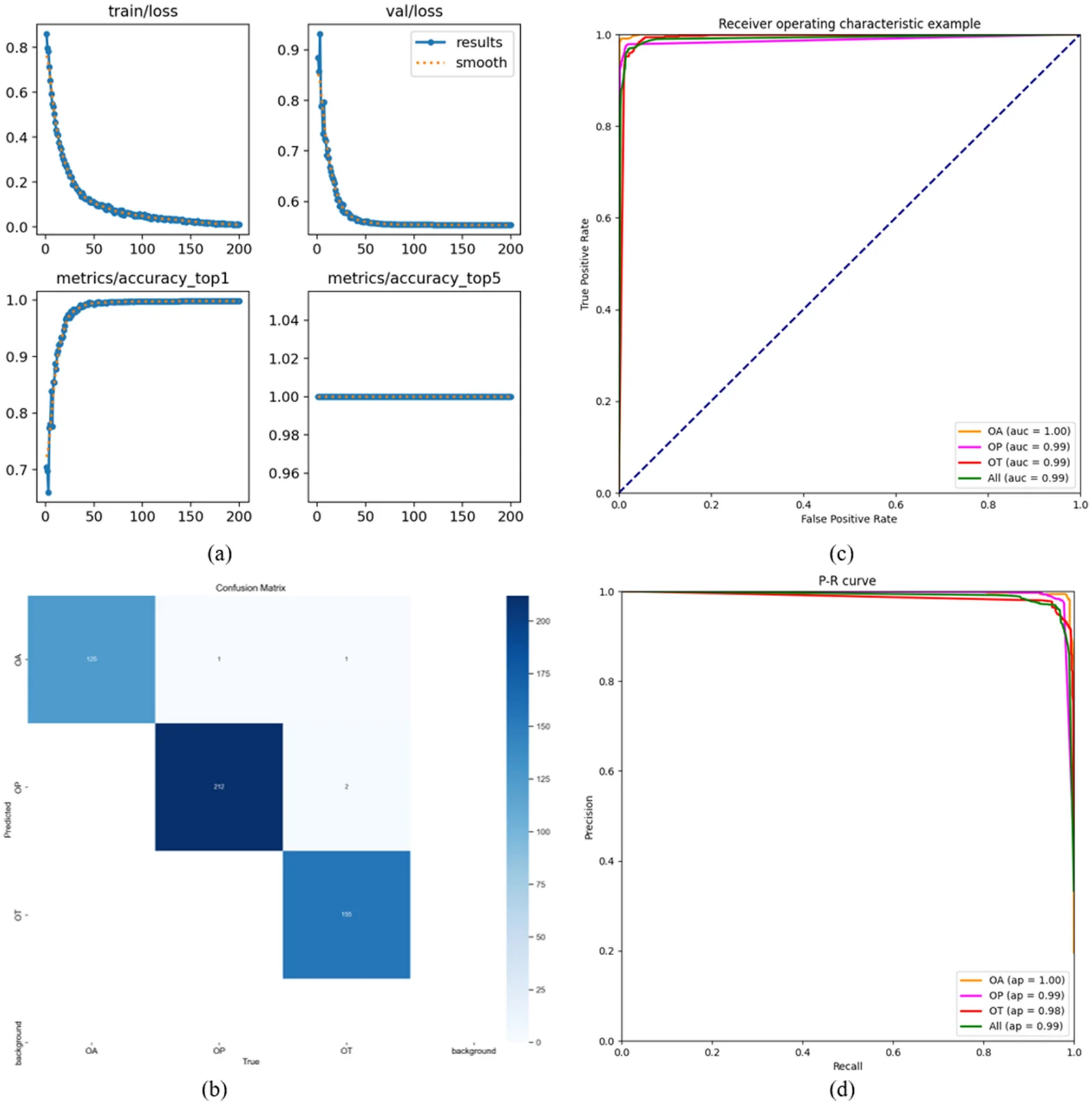
Training result curve graph.

The proposed model for classifying fetal head position in intrapartum ultrasound images is based on the following parameters: the optimizer is SGD, the initial learning rate is 0.01, batch size is 16, and image size is 512. The data of the training set is enhanced, and the improved model is used for 200 rounds of training, and the results are shown in the Table 4 and Figure 4. The classification accuracy of the model was 0.962 for ACC, 0.987 for PR-AUC, 0.991 for ROC-AUC, and 0.941 for kappa consistency test. From the results, the classification model showed very good performance, and the model prediction results were almost perfectly consistent with the actual classification.

**Table 4:** Model training results.

PR-AUC	ROC-AUC	ACC	kappa
0.8899	0.9401	0.8961	0.8434

When the model predicts a picture, it obtains a set of confidence scores, and selects the category corresponding to the highest confidence score as the prediction result of the picture. The model predicts each picture and gives the prediction category and confidence score. The prediction results with confidence score less than 0.9 are discarded. The number of pictures with different prediction results in the video is counted, and the one with the largest number of pictures is regarded as the final prediction result. We collected a total of 109 intrapartum ultrasound videos, which were scanned only from head to toe in the central axis of the human body, each video was about 8–10s long, and each video contained only one fetal head position. To verify the effectiveness of our model in classifying the fetal head position of intrapartum ultrasound videos, we compared our model with other different models, and at the same time, we also invited a midwife and a sonographer to classify the videos as well. The number of videos correctly classified by our model and different models and doctors is shown in Table 2. It can be seen that the number of videos accurately classified by our model is the largest among all the models. At the same time, our correct number can exceed that of midwives and close to that of sonographers.

### Comparative experiments

We compare the performance of our proposed model against several state-of-the-art classification models, including EfficientNet (27), DenseNet (28), ResNet (29), ConvNeXt (30), YOLOv5-cls (31), and YOLOv8-cls (32). In order to ensure the fairness of the comparison experiment, all the models in the comparison adopted a completely consistent input preprocessing process. All the input images were uniformly scaled to 512 × 512 by bilinear interpolation before entering the network, and all the models were trained from scratch without external pre-training weights. The training parameters were configured with an initial learning rate of 0.01, momentum of 0.9, weight decay of 1e-4, 200 epochs, batch size of 16, and SGD optimizer.

The trained models were then evaluated on the test set, and the quantitative results are summarized in Table 1. As evidenced in Table 1, our model achieves the best performance across all four evaluation metrics. Specifically, it outperforms the second-best model (YOLOv8-cls) by 1.55 percentage points in Accuracy and improves the Kappa coefficient by 0.0267, demonstrating its superior classification capability.

### Ablation experiments

To validate the effectiveness of each individual module in our proposed model, we conducted a series of ablation studies. We created several model variants by removing one or more modules from the full model, while keeping the backbone network and all hyperparameters constant. The model variant without any of our proposed modules serves as the baseline. All model variants were trained under the same settings as in the comparative experiments.

The results are presented in Table 5. It is evident that removing any single module leads to a significant performance drop across all metrics, confirming the positive contribution of each component. For example, the absence of the CBAM module results in a 5.89 percentage point decrease in Accuracy. Moreover, the full model, which integrates all modules, consistently outperforms any partial configuration, indicating that the modules work synergistically to achieve optimal performance.

**Table 5:** Comparison of results from different models.

Modules				Metrics			
CBAM	PSA	ECA	AIFI	PR-AUC	ROC-AUC	ACC	Kappa
X	X	X	X	0.8719	0.9282	0.8806	0.8169
X	✓	✓	✓	0.8041	0.8791	0.7925	0.6873
✓	X	✓	✓	0.8381	0.9046	0.8372	0.7527
✓	✓	X	✓	0.8582	0.9222	0.8633	0.7925
✓	✓	✓	X	0.8431	0.9092	0.8432	0.7626
**✓**	**✓**	**✓**	**✓**	**0.8899**	**0.9401**	**0.8961**	**0.8434**

The bold value represents the best result in the comparison.

**Figure 5:**
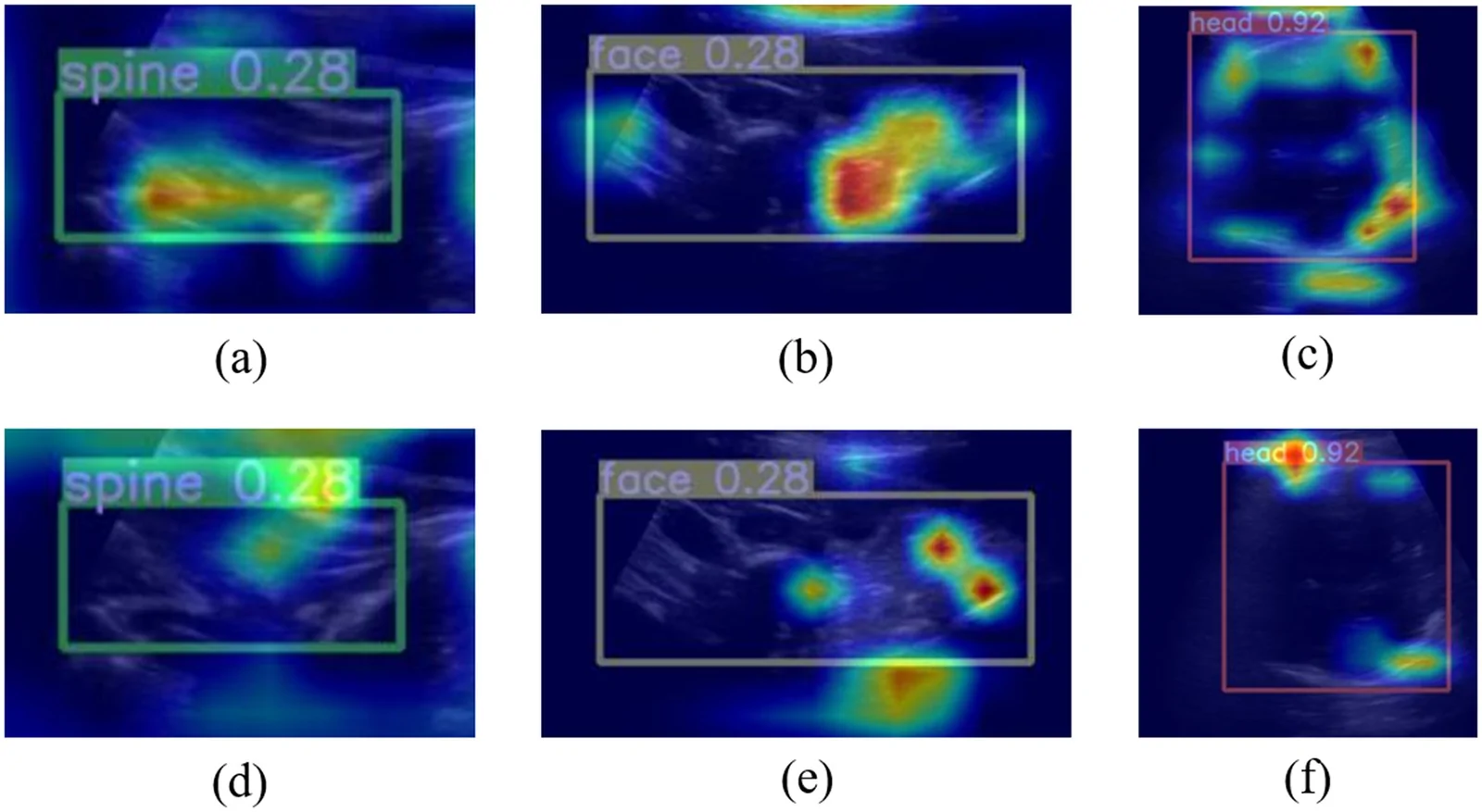
Heat map - the area of concern of the model. The top row is our improved model, and the bottom row is the baseline model. It can be seen that our improved model ca0.n better focus on the key organs in the fetal head position. **(A, D)** are OA, **(B, E)** are OP, and **(C, F)** are OT.

As shown in Figure 5, The top row is our improved model, and the bottom row is the baseline model, the model with attention mechanism can better focus on the location of key organs in the picture.

## Discussion

In this study, we proposed FHP-ClsNet, a deep learning model designed to classify fetal head positions (occiput anterior, posterior, and transverse) from intrapartum ultrasound images. Building upon the YOLOv8-cls architecture, we incorporated the CBAM, ECA, and PSA attention mechanisms to enhance feature representation, alongside the AIFI module to refine high-level feature interactions via self-attention. Experimental results demonstrate that these modifications yielded a significant performance improvement, with the model achieving an accuracy of 89.61% and a kappa coefficient of 0.8434 on the test set.

While previous studies, such as the work of Ghi et al. (19) and Dall'Asta et al. (20), have established the feasibility of deep learning for intrapartum fetal head position classification, these investigations primarily employed standard convolutional neural network (CNN) architectures or customized classifiers. Such approaches often lack fundamental innovations at the architectural level concerning feature interdependence. In contrast, our study focused on exploring the synergistic effects of advanced attention mechanisms within the backbone network. To ensure a fair evaluation of the performance gains derived from our architectural improvements, we benchmarked FHP-ClsNet against a range of representative general image classification architectures, including ResNet, DenseNet, EfficientNet, and ConvNeXt. This comparative strategy directly demonstrates the superiority of our proposed components in processing the complex texture and low contrast inherent in ultrasound imagery.

It is important to acknowledge that while our model performed robustly on single-institution static image classification, a comparative analysis with other studies reveals a common trade-off in medical imaging analysis. Studies utilizing simpler architectures but trained on broader, multicenter datasets (e.g., Ramirez Zegarra et al. (22) have reported higher absolute performance metrics. This observation underscores the principle that while complex architectures possess stronger theoretical feature extraction capabilities, their full potential is often contingent on large-scale and diverse training data. The dataset utilized in this study was derived from a single medical center, which may inherently limit the generalizability of the model. Consequently, future work should prioritize expanding the dataset to include a wider variety of ultrasound equipment and patient demographics to validate the robustness of our complex architecture in real-world multicenter scenarios.

Furthermore, although our model showed high accuracy in still image classification, its performance on video sequences was comparatively lower. This suggests a loss of temporal contextual information when processing videos frame-by-frame without specific spatiotemporal modeling. In clinical practice, the dynamic assessment of fetal head descent and rotation is crucial. Therefore, future research should focus on incorporating temporal modeling techniques, such as recurrent neural networks (RNNs) or graph neural networks (GNNs), to better capture the sequential information in ultrasound videos.

## Conclusion

This study aimed to develop an accurate deep learning model, FHP-ClsNet, for automated classification of fetal head positions from intrapartum ultrasound images. By integrating multiple attention mechanisms (CBAM, ECA, PSA) and an AIFI feature interaction module into the YOLOv8 architecture, the proposed model demonstrated strong performance in distinguishing between occiput anterior, posterior, and transverse positions. The results confirm that the integration of attention modules significantly enhances feature representation and classification accuracy compared to baseline models. The main contribution of this work lies in providing a standardized, efficient tool to assist sonographers and midwives in fetal head position assessment, reducing reliance on subjective manual interpretation. This has practical implications for improving labor management and supporting clinical decision-making, particularly in settings with limited expertise. However, the study has certain limitations: the model's performance on video sequences was lower than on static images, indicating a loss of temporal context, and its generalizability across different ultrasound devices and populations requires further validation. Future research should focus on incorporating temporal modeling techniques, such as graph neural networks or recurrent architectures, to better capture sequential information in ultrasound videos. Expanding the dataset with more diverse clinical samples will also be essential to enhance robustness and applicability. In summary, FHP-ClsNet represents a meaningful step toward automated intrapartum ultrasound analysis, with potential to increase diagnostic efficiency and consistency in obstetric practice.

## Data Availability

The datasets presented in this article are not readily available because research data are not shared. Requests to access the datasets should be directed to Peizhong Liu, pzliu@hqu.edu.cn.
